# IGF-1C hydrogel improves the therapeutic effects of MSCs on colitis in mice through PGE_2_-mediated M2 macrophage polarization

**DOI:** 10.7150/thno.45434

**Published:** 2020-06-19

**Authors:** Xiaocang Cao, Liyun Duan, Huixing Hou, Yue Liu, Shang Chen, Shuaiqiang Zhang, Yuanyuan Liu, Chen Wang, Xin Qi, Na Liu, Zhibo Han, Dekui Zhang, Zhong-Chao Han, Zhikun Guo, Qiang Zhao, Zongjin Li

**Affiliations:** 1 Department of Hepato-Gastroenterology, Tianjin Medical University General Hospital, Tianjin Medical University, Tianjin, China.; 2 Nankai University School of Medicine, Tianjin, China.; 3 Department of Cardiology, Tianjin Union Medical Center, Nankai University Affiliated Hospital, Tianjin, China.; 4 Tianjin Key Laboratory of Engineering Technologies for Cell Pharmaceutical, National Engineering Research Center of Cell Products, AmCellGene Co., Ltd., Tianjin China.; 5 Jiangxi Engineering Research Center for Stem Cell, Shangrao, Jiangxi, China.; 6 Gastroenterology Department, Lanzhou University Second Hospital, Gansu, China.; 7 Henan Key Laboratory of Medical Tissue Regeneration, Xinxiang Medical University, Xinxiang, China.; 8 The Key Laboratory of Bioactive Materials, Ministry of Education, the College of Life Science, Nankai University, Tianjin, China.; 9 State Key Laboratory of Kidney Diseases, Chinese PLA General Hospital, Beijing, China.

**Keywords:** Inflammatory bowel disease (IBD), Mesenchymal stem cells (MSCs), M2 macrophage polarization, IGF-1C hydrogel, Prostaglandin E_2_ (PGE_2_)

## Abstract

**Background:** Mesenchymal stem cell (MSC)-based therapies hold great promise for the treatment of inflammatory bowel disease (IBD). In order to optimize and maximize the therapeutic benefits of MSCs, we investigated whether cotransplantation of a chitosan (CS)-based injectable hydrogel with immobilized IGF-1 C domain peptide (CS-IGF-1C) and human placenta-derived MSCs (hP-MSCs) could ameliorate colitis in mice.

**Methods:** IGF-1C hydrogel was generated by immobilizing IGF-1C to CS hydrogel. Colitis was induced by 2,4,6-trinitrobenzene sulfonic acid (TNBS) in mice. We initially applied hP-MSCs and CS-IGF-1C hydrogel for the treatment of colitis by *in situ* injection, and molecular imaging methods were used for real-time imaging of reactive oxygen species (ROS) and tracking of transplanted hP-MSCs by bioluminescence imaging (BLI). Furthermore, the effects of CS-IGF-1C hydrogel on prostaglandin E_2_ (PGE_2_) secretion of hP-MSCs and polarization of M2 macrophages were investigated as well.

**Results:** The CS-IGF-1C hydrogel significantly increased hP-MSC proliferation and promoted the production of PGE_2_ from hP-MSCs *in vitro*. Moreover, *in vivo* studies indicated that the CS-IGF-1C hydrogel promoted hP-MSC survival as visualized by BLI and markedly alleviated mouse colitis, which was possibly mediated by hP-MSC production of PGE_2_ and interleukin-10 (IL-10) production by polarized M2 macrophages.

**Conclusions:** The CS-IGF-1C hydrogel improved the engraftment of transplanted hP-MSCs, ameliorated inflammatory responses, and further promoted the functional and structural recovery of colitis through PGE_2_-mediated M2 macrophage polarization. Molecular imaging approaches and therapeutic strategies for hydrogel application provide a versatile platform for exploring the promising therapeutic potential of MSCs in the treatment of IBD.

## Introduction

Based on their immunomodulatory and tissue regenerative potential, mesenchymal stem cells (MSCs) have been proposed as promising candidates for the treatment of inflammatory bowel disease (IBD) 1-3. There is increasing evidence that MSCs can regulate the biological activities of a variety of immune cells, such as T cells, B cells, NK cells, neutrophils and macrophages 4. At present, the immunomodulatory mechanism of MSCs is still not fully elucidated, but it is generally believed that MSCs exert their immunosuppressive function mainly through intercellular contact and secretion of soluble factors, such as transforming growth factor-β1 (TGF-β1), hepatocyte growth factor (HGF), indoleamine 2,3-dioxygenase (IDO), nitric oxide (NO), and prostaglandin E_2_ (PGE_2_) 5, 6. Compared with MSCs derived from other tissues, MSCs from the placenta possess better immunoregulatory properties 7.

The largest population of macrophages in the body resides in the gastrointestinal tract, which plays pivotal roles in the maintenance of mucosal homeostasis and is also an important component of protective immunity 2. M1 macrophages can release inflammatory cytokines to induce an inflammatory response. However, M2 macrophages can secrete interleukin-10 (IL-10) to exert anti-inflammatory effects 8. Overall, macrophages play dual roles in regulating inflammation via the M1 phenotype and can switch to an M2 phenotype. MSCs could increase the secretion of anti-inflammatory factors, including IL-10, IL-1 receptor antagonist (IL-1RA) and arginase (Arg)-1, and induce macrophages to switch from proinflammatory phenotypes (M1) to regulatory phenotypes (M2), which could inhibit inflammatory responses and promote tissue repair and regeneration in IBD 9-11. Applications of MSCs in experimental colitis have confirmed that PGE_2_ secreted by MSCs is involved in regulating the secretory function and phenotypic transformation of macrophages 12, 13.

Transplantation of MSCs has been considered a good therapeutic for the treatment of several immune disorders, including IBD 13, 14. However, the outcome of stem cell therapy depends on the number of surviving cells after transplantation 15. Poor cell engraftment and retention after transplantation are the main factors limiting current stem cell-based therapy 14. It has been shown that engineered matrices and growth factors mimicking native stem cell microenvironments provide supportive niches for transplanted stem cells and further enhance the therapeutic effects of stem cells 14, 16. Our previous data revealed that a bioactive hydrogel immobilizing the C domain peptide of insulin-like growth factor 1 (IGF-1C) on chitosan protected the delivered adipose-derived MSCs, promoted stem cell survival, maintained a long and stable therapeutic action, and facilitated MSC paracrine and anti-inflammatory effects 14, 17. In addition, recent studies have demonstrated that insulin-like growth factor-1 (IGF-1) could shape the macrophage-activation phenotype 18, 19.

In this study, we hypothesized that the IGF-1C hydrogel could improve the therapeutic effects of MSCs in experimental colitis through PGE_2_-mediated M2 macrophage polarization. We cotransplanted CS-IGF-1C hydrogel with hP-MSCs into the topical site of trinitrobenzene sulfonic acid (TNBS)-induced colitis to detect the therapeutic effects. In parallel, we explored whether cotransplanted CS-IGF-1C hydrogel with hP-MSCs ameliorated colitis by promoting the release of PGE_2_ to promote the polarization of M2 macrophages, accompanied by the production of IL-10, reducing the level of M1 macrophages and proinflammatory cytokines.

## Materials and Methods

### Cell culture

HP-MSCs were isolated from the human placenta based on previously reported methods 20. HP-MSCs were cultured in complete Dulbecco's modified Eagle's medium (DMEM)/F12 (Gibco, Grand Island, NY) consisting of 10% fetal bovine serum (FBS, HyClone, Australia), 1% L-glutamine (Gibco), 1% nonessential amino acids (Gibco) and 1% penicillin-streptomycin (Gibco). To track transplanted cells *in vivo*, hP-MSCs of passage 3 were transfected with a self-inactivating lentiviral vector that carried the ubiquitin promoter driving firefly luciferase and enhanced the green fluorescent protein (Fluc-eGFP) double fusion (DF) reporter gene as previously described 11.

### Isolation of mouse peritoneal macrophages

Peritoneal macrophages were extracted from the mouse abdominal cavity as previously reported 21. Briefly, 3 ml of 3% sterilized thioglycolate (Sigma, USA) was injected into the abdominal cavity of male BALB/c mice (22 ± 3 g). After four days, the mice were killed, followed by peritoneal lavage with 8 mL of PBS containing 3% FBS. Cell suspensions were centrifuged at 1500 rpm for 8 min, and the supernatant was discarded. Complete DMEM (Gibco) containing 10% FBS (HyClone), 1% L-glutamine (Gibco), 1% nonessential amino acids (Gibco) and 1% penicillin-streptomycin (Gibco) was used to resuspend the cell pellet, and then the cells were seeded in a cell culture dish and incubated for 2 h. Nonadherent cells were washed off with PBS, and adherent peritoneal macrophages were then rested overnight at 37 ºC with 5% CO_2_. Lipopolysaccharide (LPS) was used to stimulate the cells for 12 h, and PGE_2_ or conditioned medium of hP-MSCs cultured on CS-IGF-1C hydrogel-coated plates was added for 24 h.

### Cell proliferation assay

The preparation and characterization of CS-IGF-IC hydrogel was reported in a previous study 14. The cell counting kit-8 (Dojindo Molecular Technologies, Rockville, MD) assay and cell bioluminescence imaging (BLI) were used to determine the optimal concentration of CS-IGF-1C hydrogel for hP-MSC proliferation. Ninety-six-well plates were coated with different concentrations of CS-IGF-1C hydrogel (50, 100, 200, 400, and 800 μg/mL), and 1×10^4^ hP-MSCs were added per well. After 24 h, the absorbance at 450 nm was measured in an automated microplate absorbance reader, and the BLI signal was measured using the IVIS Lumina Imaging System 22.

To evaluate the proliferation of hP-MSCs cultured under different conditions (noncoated, CS hydrogel-coated and CS-IGF-IC hydrogel-coated plates), 3×10^3^ hP-MSCs were added to the wells of 96-well plates, and BLI assays were carried out after 24, 48, 72, and 96 h. In addition, the proliferation-related gene expression of hP-MSCs cultured under different conditions (noncoated, CS hydrogel-coated, and CS-IGF-IC hydrogel-coated plates) was analyzed by RT-PCR at 72 h.

### Enzyme-linked immunosorbent assay

To determine the PGE_2_ concentrations in cell-free supernatants collected from different conditions and fresh colon tissues, enzyme-linked immunosorbent assays (ELISAs) were carried out. hP-MSCs were cultured on CS-IGF-1C hydrogel-coated, CS hydrogel-coated, or noncoated plates for 48 h, and cell-free supernatants of each well were harvested. The colon tissues were collected after different treatments. PGE_2_ ELISA kits (Abcam, Cayman Chemicals) were used strictly following the manufacturer's instructions.

### Induction and treatment of colitis

Eight-week-old male BALB/c mice were purchased from the Laboratory Animal Center of the Academy of Military Medical Science (Beijing, China). All experiments were conducted in conformity with institutional guidelines for the Nankai University Animal Care and Use Committee, which conform to the National Institutes of Health (NIH) Guide for the Care and Use of Laboratory Animals. Colitis was induced by TNBS (Sigma-Aldrich) according to the method described previously 23. Briefly, BALB/c mice were anesthetized by intraperitoneal injection of 2.5% avertin (Sigma-Aldrich) at a dose of 240 mg/kg, and then the TNBS (100 mg/kg) dissolved in 50% ethanol was slowly injected into the rectum via a catheter equipped with a 1 mL syringe. Mice were held in a vertical position for 1 min to ensure the distribution of the reagent within the entire colon and cecum. Control group mice were administered only 50% ethanol solution.

After 24 h of TNBS enema, the induction of colitis was confirmed by surgical inspection, and 1×10^6^ hP-MSCs (Fluc-GFP) were gently injected into three sites of the injured colon mesangial margin through the connection between the mesentery and intestinal wall at 30 μL total volume suspended in PBS, CS hydrogel, or CS-IGF-1C hydrogel (n=10 for each group). The injection of PBS served as a control. Sham-operated animals were subjected to the same surgical procedure without colitis or cell/hydrogel transplantation. The schematic illustration of laparotomy was described in **Figure S1**.

### Bioluminescence imaging

Fluc activity within different cell numbers and the fate of transplanted cells were confirmed using a Xenogen IVIS Lumina Imaging System (Xenogen Corporation, Hopkinto, MA) as described previously 13. In short, animals were imaged for 1-10 min using the IVIS Lumina Imaging System after intraperitoneal injection of the reporter probe D-luciferin (150 mg luciferin/kg body wt, Calipers, USA). The signal intensity was analyzed by utilizing the live image software (Xenogen Corporation). The average radiance of the peak BLI signal was quantified from a fixed-area region of interest (ROI) over the abdomen. In addition, to examine the severity of colitis, reactive oxygen species (ROS) were detected via intraperitoneal injection of luminol (10 mg/kg, 5-amino-2,3-dihydro-1,4-phthalazinedione, Yeasen, USA) using the IVIS Lumina Imaging System. The luminol stock solutions were prepared in normal saline prior to injection 24.

### Quantitative RT-PCR

Total RNA was isolated from the cells or colons by TRIzol reagent (Invitrogen, Grand Island, NY) according to the manufacturer's directions. First-strand cDNA was synthesized using oligo dT primers with reverse transcriptase (TransGen Biotech, China). Subsequently, real-time PCR was performed on an Opticon® System (Bio-Rad, Hercules, CA) in 10 µL reaction volumes. The mRNA expression levels were quantified using the Trans Start Green qPCR SuperMix Kit (TransGen Biotech, China). The 2^-ΔΔCt^ method was used to determine the relative mRNA fold changes. Primers are listed in **Table S1**.

### Histochemical and immunofluorescence staining

To investigate the inflammatory response of colitis, animals were euthanized on day 3. The isolated representative colon tissues were immediately washed in PBS and fixed with 4% paraformaldehyde. Hematoxylin eosin (HE) staining and immunohistochemical staining were performed to assess colonic inflammation and damage according to the histopathologic grading system of MacPherson and Pfeiffe 25. For immunofluorescence staining, sections were incubated with primary antibodies against F4/80 (Abcam), CD206 (Abcam), iNOS (inducible nitric oxide synthase, Abcam), EpCAM (Abcam) and GFP (Santa Cruz) overnight at 4 ºC and then incubated with Alexa Fluor 488 and 594 (Invitrogen, Grand Island, NY). Cell nuclei were counterstained with 4,6-diamidino-2-phenylindole (DAPI; Southern Biotech, Birmingham, AL). The number of positive cells was counted in 15 randomly selected areas using a fluorescence microscope (CD206 and iNOS) as previously described 26.

To determine whether the polarization of M2 macrophages and the decrease in M1 macrophages were related to PGE_2_, the supernatants of hP-MSCs/CS-IGF-1C were collected using the same method described above in 6-well plates, and the supernatants and 1 μM PGE_2_ or PF (prostaglandin EP2 receptor antagonist, PF-04418948) were added to different wells of macrophages. After 12 h, the cells were assayed for CD206 and iNOS (1:200, rabbit anti mouse, Santa Cruz, USA) by immunofluorescence.

### Myeloperoxidase activity assay

Measurement of myeloperoxidase (MPO) activity was used to monitor neutrophil infiltration and was evaluated by the MPO kit (Jiancheng, Nanjing, China) according to the manufacturer's instructions 27.

### Western blot assay

To detect the changes in M1 and M2 macrophages in colon tissue after hP-MSCs were cotransplanted with CS-IGF-1C hydrogel. Colon tissue was lysed on ice in radioimmunoprecipitation assay (RIPA) buffer, and the protein was quantified by the BCA Protein Assay Kit (Promega), separated by 10% SDS-PAGE, and transferred onto polyvinylidene fluoride membranes (Millipore, Darmstadt, Germany). The primary antibodies used for western blot analysis were CD206 and iNOS (1:1000, Santa Cruz, CA) and IL-10 (1:1000, Abcam, Cambridge, UK). β-Actin (1:1000, Santa Cruz Biotechnology, CA) was used as an internal control.

### Statistical analyses

Statistical analyses were performed using GraphPad Prism 5.0 software (GraphPad Software Inc., San Diego, CA). One- or two-way repeated-measures ANOVA and two-tailed t test were used. All the treatments were further compared through post test to determine the differences among groups. Differences were considered significant at *P* values <0.05.

## Results

### Characterization of hP-MSCs

For monitoring transplanted cells *in vivo*, cell lines with double fusion reporter genes (Fluc-GFP) were developed **(Figure S2A)**. Transduction of hP-MSCs with double fusion genes did not affect the cell morphology, and the cells were strongly positive for green fluorescence protein (GFP)** (Figure S2B)**. In addition, BLI revealed a linear correlation between the cell number and luciferase signal **(Figure S2C-D)**.

**Figure 1:**
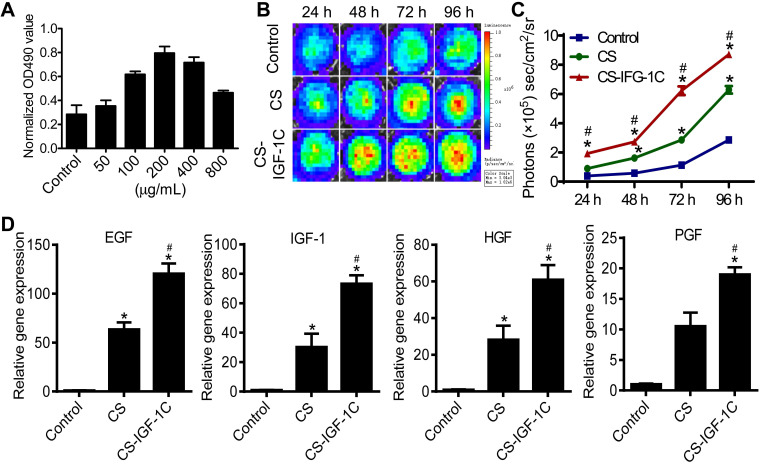
**Biocompatibility of CS-IGF-1C hydrogel. (A)** Cell proliferation assay of hP-MSCs under different concentrations of CS-IGF-1C hydrogel. (**B**) Cell proliferation of hP-MSCs cultured on plates coated with CS-IGF-1C hydrogel was promoted. The signal activity is expressed as photons/s per cm^2^ per steradian (sr). (**C**) Quantitative analysis of BLI signal intensity. **(D)** Proliferation-related gene expression of EGF, IGF-1, HGF, and PGF in hP-MSCs cultured on plates coated with CS-IGF-1C, CS hydrogel or noncoated plates. Data are expressed as the mean ± SD.^ *^P<0.05 versus control; ^#^*P*<0.05 versus CS hydrogel. All experiments were performed in triplicate.

To examine the bioactivities of this hydrogel, a cell proliferation assay was carried out. The results of the Cell Counting Kit 8 (CCK-8) and BLI indicated that the optimal concentration of CS-IGF-1C hydrogel for cell proliferation was 200 μg/mL **(Figure 1A and Figure S3A)**. BLI also revealed the enhanced proliferation of hP-MSCs cultured on CS-IGF-1C hydrogel-coated plates compared to noncoated and CS-coated plates **(Figure 1B-C)**. Moreover, proliferation-related genes, including epithelial growth factor (EGF), insulin-like growth factor-1 (IGF-1), hepatocyte growth factor (HGF), placental growth factor (PGF), platelet-derived growth factor (PDGF), and vascular endothelial growth factor (VEGF), were analyzed by real-time PCR. The results showed that all these genes were upregulated in hP-MSCs cultured on CS-IGF-1C hydrogel-coated plates compared with those cultured on noncoated plates and CS hydrogel-coated plates** (Figure 1D, Figure S3B).** The results of RT-PCR analysis showed that PGE_2_-related gene expression (COX-1 and COX-2) was upregulated in hP-MSCs cultured on CS-IGF-1C hydrogel-coated plates **(Figure S3C)**. Next, to determine if *in vitro* exposure to hydrogel will affect the phenotypic characteristic of hP-MSCs. After cultured on chitosan or CS-IGF-1C hydrogel coated plates for 3 days, hP-MSCs maintained the expression of surface markers (CD73, CD90, CD105) typically found on MSCs **(Figure S4)**. The results indicate that the phenotypic of hP-MSCs were not influenced by CS-IGF-1C or chitosan hydrogel in *in vitro* culture.

### Enhanced engraftment of hP-MSCs

**Figure 2:**
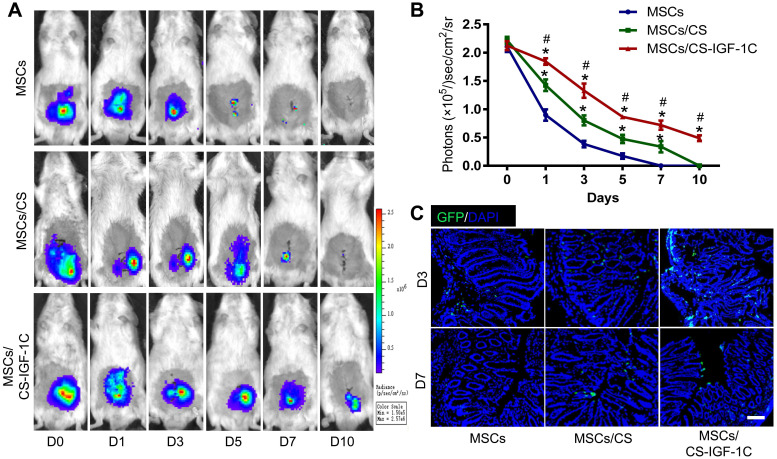
** CS-IGF-1C hydrogel enhanced hP-MSC survival *in vivo*.** (**A**) Cell fate of hP-MSCs after transplantation with CS hydrogel, CS-IGF-1C hydrogel or PBS in a TNBS-induced mouse colitis model. (**B**) Quantitative analysis of BLI signals. Data are expressed as the mean ± SD. n=10. ^*^P<0.05 versus hP-MSCs;^ #^*P*<0.05 versus hP-MSCs/CS hydrogel. (**C**) Survival of hP-MSCs (GFP, green) cotransplanted with CS hydrogel or CS-IGF-1C hydrogel. Cell nuclei were counterstained with DAPI (blue). Scale bars, 100 µm. hP-MSCs/CS, hP-MSCs cotransplanted with CS hydrogel; hP-MSCs/CS-IGF-1C, hP-MSCs cotransplanted with CS-IGF-1C hydrogel. All experiments were performed in triplicate.

After various treatments (hP-MSCs, hP-MSCs cotransplanted with CS hydrogel, and hP-MSCs cotransplanted with CS-IGF-1C hydrogel), the survival of hP-MSCs was monitored longitudinally by BLI at days 0, 1, 3, 5, 7, and 10. The result of BLI analysis exhibited robust signals from the colon region at day 0 after topical delivery of hP-MSCs in all groups, which indicated successful hP-MSC transplantation **(Figure 2A-B)**. Serial BLI of the same animals demonstrated ameliorated cell engraftment by CS hydrogel application, and CS-IGF-1C hydrogel further increased this effect, suggesting that CS-IGF-1C hydrogel could augment cell survival and therefore increase the therapeutic potential of hP-MSCs. The results of immunofluorescence staining also confirmed that CS-IGF-1C promoted the survival of hP-MSCs (**Figure 2C**).

### Enhanced protective effects of hP-MSCs with CS-IGF-1C hydrogel

**Figure 3:**
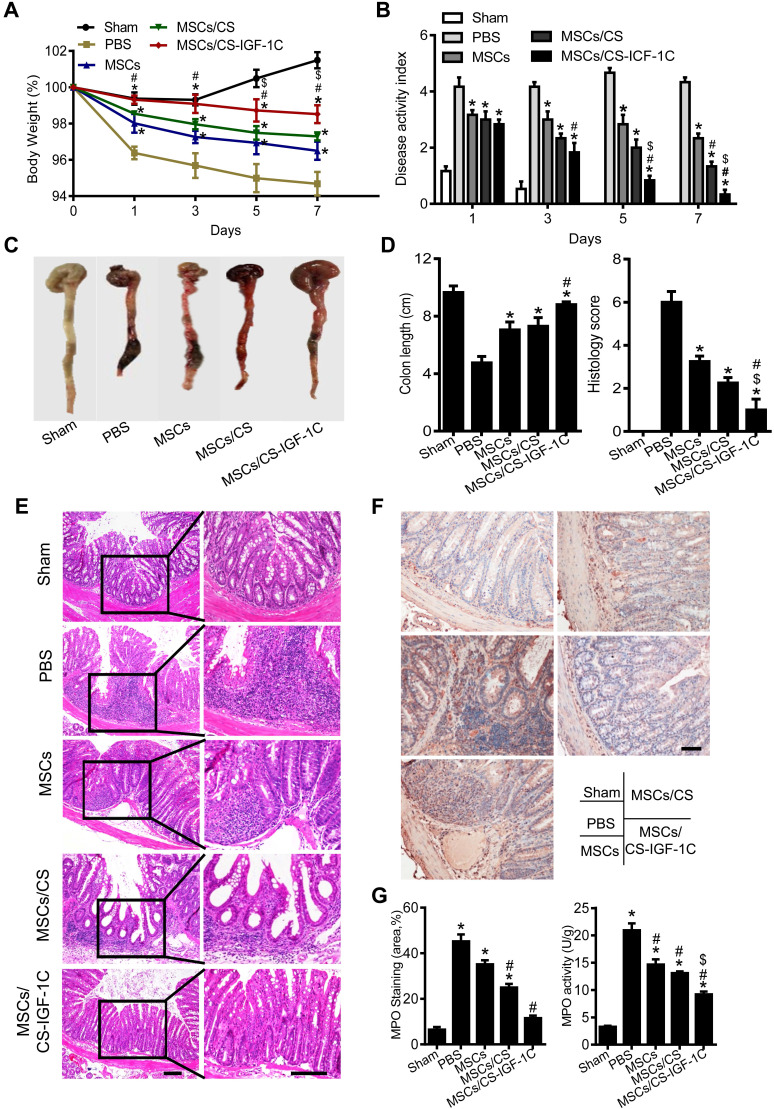
** CS-IGF-1C hydrogel facilitated the therapeutic effect of hP-MSCs.** (**A**) Percentage of body weight loss after treatment. (**B**) The DAI score at day 7. The weight loss index, bloody stool index, and stool consistency index were calculated. (**C**) Macroscopic images of colonic tissues at day 3 after treatment. (**D**) Quantification of the length of a representative inflamed colon. Data are expressed as the mean ± SD. n=10. ^*^P<0.05 versus PBS; ^#^*P*<0.05 versus hP-MSCs; ^$^*P*<0.05 versus hP-MSCs/CS. (**E**) Representative histological sections of mouse colons stained with H&E. Scale bar, 100 µm. (**F**) Representative MPO immunohistochemical staining. Positive cells were stained yellow or brownish‑yellow, and the distribution of positive cells in the colon was mainly localized in the lamina propria of the mucosa and scattered around the intestinal gland. Scale bar, 100 µm. (**G**) Myeloperoxidase (MPO) immunohistochemistry score. MPO activity was measured in homogenates of the distal colon to evaluate the number of neutrophils. hP-MSCs/CS, hP-MSCs cotransplanted with CS hydrogel; hP-MSCs/CS-IGF-1C, hP-MSCs cotransplanted with CS-IGF-1C hydrogel. All experiments were performed in triplicate.

After administrating TNBS via rectal enema for 24 h, the hP-MSCs were injected into the inflammatory colon *in situ* by laparotomy. Weight change, bloody diarrhea, and lassitude (disease activity index, DAI) were measured and observed on days 1, 3, 5 and 7 after hP-MSC administration, and histological examination was performed. The results indicated that hP-MSC and CS-IGF-1C hydrogel cotransplantation significantly reduced the extent of body weight loss and the DAI score compared with the PBS, free hP-MSC, hP-MSC and CS hydrogel cotransplantation groups **(Figure 3A-B)**. To further evaluate the treatment effectiveness, we sacrificed the mice by euthanasia and excised the entire colon on day 3. The results showed that transplantation of hP-MSCs significantly improved the colon length and histological score compared with the PBS group **(Figure 3C-D)**.

Histopathological staining of colon slices from colitic mice treated with PBS showed disordered mucosal architecture, inflammatory cell infiltration, crypt loss, ulceration, and epithelial cell necrosis. Although the colonic tissue from the mice of hP-MSC-transplanted groups showed thicker epithelium and less inflammatory cell infiltration within the lamina propria, the hP-MSCs cotransplanted with CS-IGF-1C hydrogel groups showed the lowest levels of inflammation **(Figure 3E)**. Meanwhile, we investigated MPO activity of the injured colon and our results shown that MPO was significantly downregulated in the MSCs/CS-IGF-1C treated group **(Figure 3F-G).** Inflammation was severe in the PGE_2_-deficient hP-MSCs cotransplanted with CS-IGF-1C hydrogel group **(Figure S5A)**. In addition, we investigated whether MSCs/CS-IGF-1C played a role in intestinal epithelial cells in the colitis mice. EpCAM immunofluorescence staining showed that the colonic tissue from the hP-MSC and CS-IGF-1C hydrogel cotransplantation groups maintained better epithelial integrity **(Figure S6A-B)**. Real-time PCR analysis of tight junction protein-related gene expression (Claudin, Occludin, and ZO-1) in the colon on day 3 after treatment. The results showed all these genes were upregulated in MSCs/CS-IGF-1C group compared with MSCs, CS and EP2/MSCs/CS-IGF-1C group (**Figure S6C).** Furthermore, treatment with hP-MSCs and CS-IGF-1C hydrogel greatly improved the survival rate of the mice **(Figure S7)**.

### Enhanced anti-inflammatory effects of hP-MSCs with CS-IGF-1C

**Figure 4:**
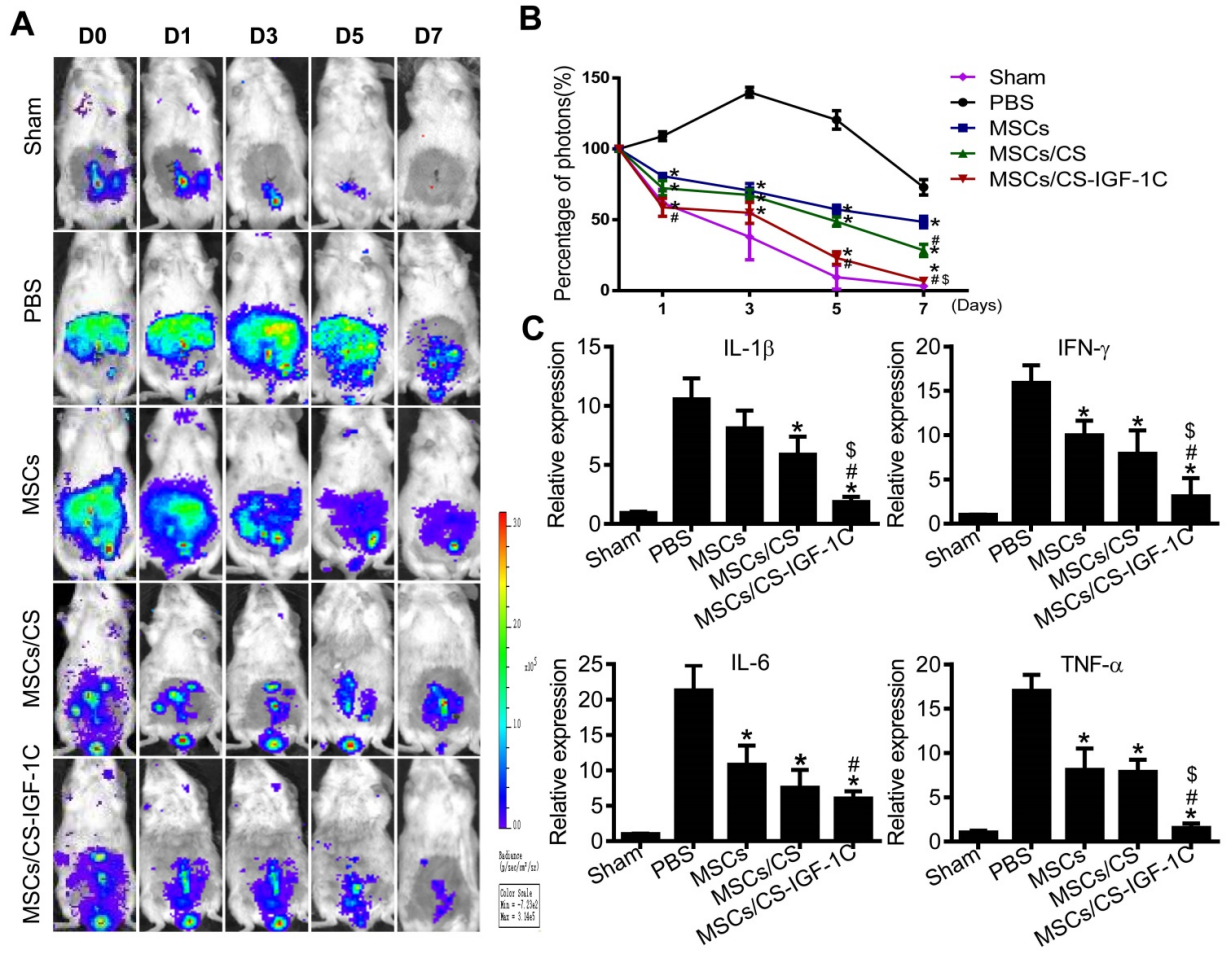
** CS-IGF-1C hydrogel enhanced the anti-inflammatory effect of hP-MSCs.** (**A**) ROS activities in mouse colitis tracked by BLI *in vivo*. (**B**) Quantitative analysis of BLI signal intensity (n=10). (**C**) The expression of proinflammatory factors (IL-1β, IFN-γ, IL-6, TNF-α) in a mouse colitis model. Data are expressed as the mean ± SD. ^*^P<0.05 versus PBS; ^#^*P*<0.05 versus hP-MSCs; ^$^*P*<0.05 versus hP-MSCs/CS. All experiments were performed in triplicate. hP-MSCs/CS, hP-MSCs cotransplanted with CS hydrogel; hP-MSCs/CS-IGF-1C, hP-MSCs cotransplanted with CS-IGF-1C hydrogel.

It has been shown that increased reactive oxygen species (ROS) contribute to the development of colitis 28. Therefore, we evaluated the level of ROS *in vivo* via BLI 29. Cotransplantation of hP-MSCs with CS-IGF-1C hydrogel significantly decreased the level of ROS** (Figure 4A-B)**. The results of RT-PCR indicated that proinflammatory factors were markedly decreased in the hP-MSCs cotransplanted with CS-IGF-1C hydrogel group compared with the other groups **(Figure 4C)**.

### Enhanced polarization of M2 macrophages by CS-IGF-1C hydrogel

**Figure 5:**
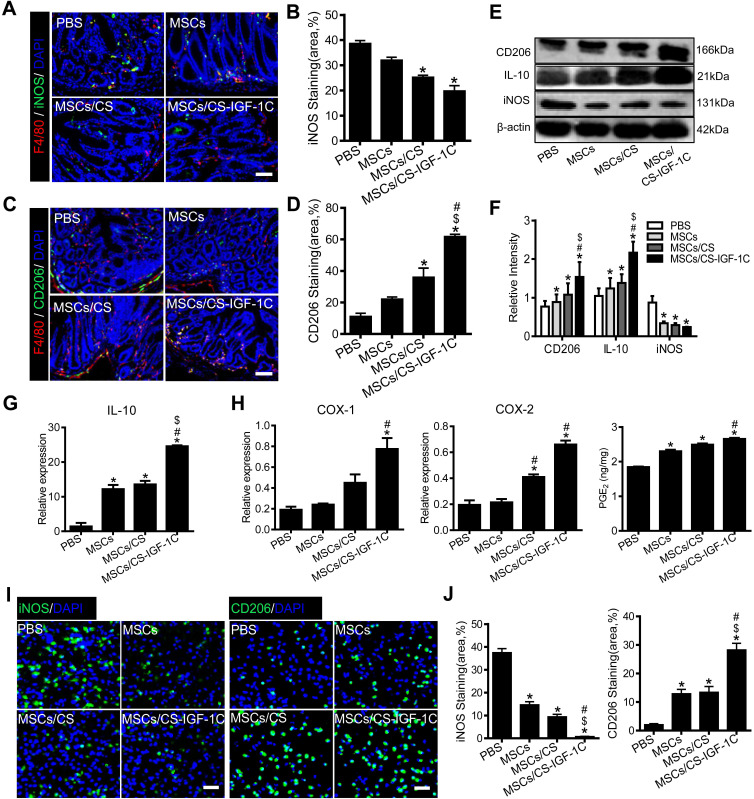
** The CS-IGF-1C hydrogel promoted hP-MSC-induced polarization of M2 macrophages.** (**A**) Representative immunofluorescence staining images of iNOS (green) and F4/80 (red) showing the accumulation of M1 macrophages on day 3. Cell nuclei were counterstained with DAPI (blue). (**B**) Quantitative analysis of iNOS-positive cells. (**C**) Representative images show CD206 (green) and F4/80 (red) immunostaining on day 3 after treatment. (**D**) Quantitative analysis of CD206-positive cells (n=10). (**E**) Western blot analysis showed the expression of CD206, iNOS and IL-10 proteins in the colon extracts on day 3 after treatment. (**F**) Relative protein intensity was normalized to β-actin. (**G**) RT-PCR analysis of IL-10 expression in the colon on day 3 after treatment. (**H**) RT-PCR analysis of PGE_2_-related gene expression (COX-1 and COX-2) in the colon on day 3 after treatment. PGE_2_ levels in homogenates of the colon on day 3. (**I**) Representative immunofluorescence staining of iNOS and CD206 (green) in peritoneal macrophages on day 3 after treatment. (**J**) Quantitative analysis of iNOS-/CD206-positive cells in peritoneal macrophages. Data are expressed as the mean ± SD, n=10. ^*^P<0.05 versus PBS; ^#^*P*<0.05 versus hP-MSCs; ^$^*P*<0.05 versus hP-MSCs/CS. Scale bar, 100 µm. hP-MSCs/CS, hP-MSCs cotransplanted with CS hydrogel; hP-MSCs/CS-IGF-1C, hP-MSCs cotransplanted with CS-IGF-1C hydrogel. All experiments were performed in triplicate.

M1 and M2 macrophages of colon tissues on day 3 after transplanting hP-MSCs were detected by immunofluorescent staining. The CS-IGF-1C hydrogel significantly decreased the number of M1 macrophages** (Figure 5A-B)** and promoted the polarization of M2 macrophages **(Figure 5C-D)**. When the EP2 receptor of PGE_2_ is inhibited, M1 macrophages increase and M2 macrophages decrease **(Figure S5B-C)**. Western blotting analysis showed that CD206 was increased significantly in the hP-MSCs cotransplanted with CS-IGF-1C hydrogel group compared to the other groups** (Figure 5E-F)**. RT-PCR results indicated that the expression of IL-10 was increased significantly in the hP-MSCs cotransplanted with CS-IGF-1C hydrogel group compared with the others** (Figure 5G and Figure S5D)**. PGE_2_-related gene expression (COX-1 and COX-2) and concentration of PGE_2_ in the injured colon were increased significantly in the hP-MSCs cotransplanted with CS-IGF-1C hydrogel group **(Figure 5H)**. Furthermore, we extracted peritoneal macrophages from each group of mice for immunofluorescence staining, and the results showed that compared with the PBS group, M2 macrophages were significantly increased in the hP-MSCs and CS-IGF-1C cotransplantation group, while M1 macrophages were significantly decreased **(Figure 5I-J)**.

### CS-IGF-1C hydrogel enhanced PGE_2_ production by hP-MSCs and subsequent IL-10 secretion by M2 macrophages* in vitro*

**Figure 6:**
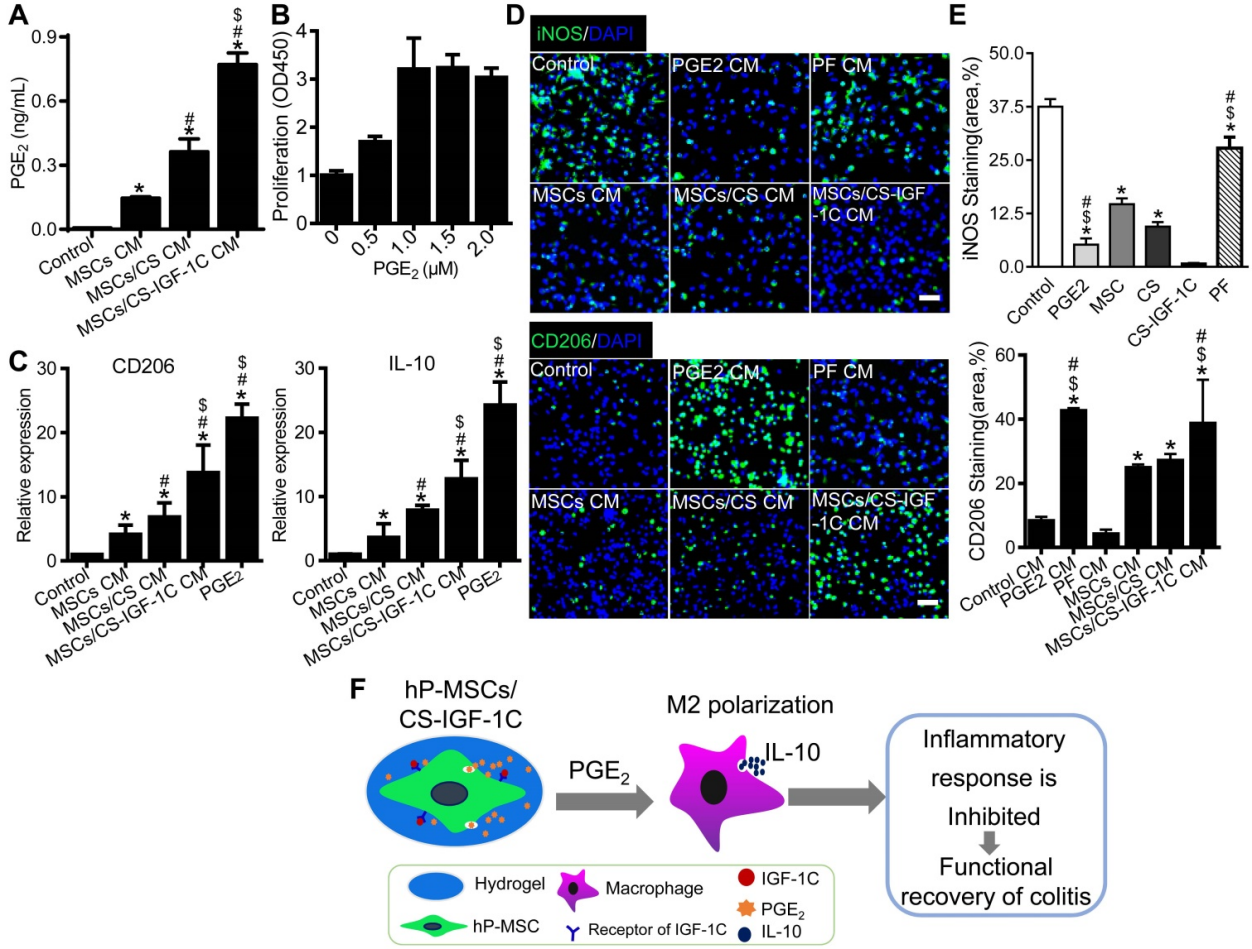
** CS-IGF-1C hydrogel enhanced the ability of hP-MSCs to secrete PGE_2_ and induce M2 macrophage polarization *in vitro.*** (**A**) The levels of PGE_2_ in the conditioned medium were detected by ELISA. (**B**) The CCK-8 assay showed the optimum concentrations of PGE_2_ for macrophage proliferation *in vitro*. (**C**) RT-PCR analysis of the gene expression of CD206 and IL-10 in macrophages after different treatments *in vitro*. (**D**) Representative images show the staining of iNOS and CD206 for peritoneal macrophages after treatment with conditioned medium for 24 h. (**E**) Quantitative analysis of iNOS- and CD206-positive cells. (**F**) Schematic diagram depicting the effects of colitis treatment after cotransplantation of hP-MSCs and CS-IGF-1C hydrogel. When cotransplanted into a mouse colitis model, the CS-IGF-1C hydrogel protected the delivered hP-MSCs and facilitated the secretion of PGE_2_, resulting in enhanced polarization of M2 macrophages, reduced inflammation, and further promotion of colon regeneration. Consequently, CS-IGF-1C hydrogel therapy leads to improved functional and structural recovery of the colon. Data are expressed as the mean ± SD. *P<0.05 versus control; ^#^*P*<0.05 versus hP-MSC CM; ^$^*P*<0.05 versus hP-MSC/CS CM. Scale bar, 100 µm. All experiments were performed in triplicate. Control, complete medium; hP-MSCs CM, conditioned medium of hP-MSCs; hP-MSCs/CS CM, conditioned medium of CS hydrogel-coated hP-MSCs; hP-MSCs/CS-IGF-1C CM, conditioned medium of CS-IGF-1C hydrogel-coated hP-MSCs.

To detect whether CS-IGF-1C can promote the secretion of PGE_2_ by hP-MSCs, the conditioned medium (CM) of the hP-MSCs cultured on CS-IGF-1C hydrogel-coated, CS hydrogel-coated, and noncoated plates was collected. The ELISA results revealed that hP-MSCs cultured on the CS-IGF-1C hydrogel produced more PGE_2_ than hP-MSCs alone or CS-treated hP-MSCs (**Figure 6A**). We further isolated abdominal macrophages from healthy mice and determined the optimal concentration of PGE_2_ promoting macrophage proliferation in a series of pilot experiments (**Figure 6B**). Macrophages were treated with PGE_2_, PF (EP2 inhibitor, PF-04418948) and CM from hP-MSCs cultured on CS-IGF-1C hydrogel-coated, CS hydrogel-coated, and noncoated plates for RT-PCR and immunofluorescence staining. PGE_2_ significantly promoted the expression of CD206 and IL-10 similar to that of hP-MSCs/CS-IGF-IC supernatants (**Figure 6C**). The CS-IGF-1C hydrogel significantly promoted the polarization of M2 macrophages and decreased the number of M1 macrophages **(Figure 6D-E)**. PGE_2_ significantly promoted M2 macrophage polarization to levels similar to those of the hP-MSC/CS-IGF-IC supernatant-treated group, but PF inhibited these effects.

## Discussion

In this study, we generated a CS-IGF-1 C hydrogel by immobilizing IGF-1C to a CS hydrogel and cotransplanted this hydrogel with hP-MSCs for the treatment of colitis. Our results revealed that CS-IGF-1C hydrogel improved the engraftment of transplanted hP-MSCs, ameliorated inflammatory responses, and promoted functional and structural recovery of colitis. These benefits can be attributed to the CS-IGF-1C hydrogel, which enhanced PGE_2_ secretion by hP-MSCs and promoted M2 macrophage polarization in the colitis model (**Figure 6F**). Moreover, the therapeutic effects of the CS-IGF-1 C hydrogel could be directly visualized by enhanced hP-MSC survival and inhibited inflammatory responses.

In this study, we injected hP-MSCs into the inflammatory colon *in situ* by laparotomy. hP-MSCs injected intravenously into the bloodstream will be nonspecifically cleared by innate immunity under physiological conditions. However, Local administration can prevent hP-MSCs from directly entering the blood circulation and greatly reduce the clearance of liver and spleen and local administration increased the concentration and duration of hP-MSCs acting on the target organ. However, the disadvantage of local administration is that the operation is relatively complex. Locally administered drugs can be administered clinically by laparoscopy and colonoscopy.

Bone marrow and adipose-derived MSCs are among the most commonly used stem cells in regenerative therapy 30. With the advantages of accessibility, high proliferation and differentiation potential, perinatal tissue-derived MSCs, such as hP-MSCs, have been widely used in research on the nervous system, cardiovascular system and autoimmune disease 30. MSC-based therapies are promising for IBD owing to their accessibility, pluripotency, and expansive potential 13. However, inconsistent results have emerged from current clinical trials involving local or systemic application 10. Future studies should focus on the pathways and mechanisms through which MSCs respond to damaged inflamed tissue in the intestine, providing vital information for improving current therapeutic strategies 31. In addition, visualizing the cellular function and molecular processes in living animals can access detailed molecular events at the molecular pathology level in certain disease stages 32, 33. In the present study, we initially applied hP-MSCs and CS-IGF-1C hydrogel to the therapy of colitis by *in situ* injection and molecular imaging methods for real-time imaging of ROS inflammation and tracking of transplanted hP-MSCs by BLI. Therefore, molecular imaging approaches and hydrogel application provide a versatile platform for exploring the therapeutic potential of MSCs in the treatment of IBD.

Stem cell therapy is partially limited by the low rate of cell retention and engraftment after transplantation for IBD 1. Growth factors, as components of the microenvironment, control stem cell fate by interactions with cells and matrices 15. Therefore, immobilization of growth factors by binding to the matrix to mimic the stem cell niche can increase stem cell survival 15. Moreover, the expression of immunoregulatory factors varies depending on the microenvironment in which MSCs are exposed 34. IGF-1 is a potent mitogenic and prosurvival factor and is implicated as a key mediator in colitis recovery 35. IGF-1 secreted by hP-MSCs may play an important role in alleviating colitis 36. Lower serum levels of IGF-1 are related to the development of colitis 37. Under inflammatory conditions, MSC-produced factors, such as PGE_2_, suppress both innate and adaptive immunity by inducing the polarization of macrophages towards an alternative phenotype 38. In the present study, we proved that the CS-IGF-1C hydrogel can enhance the therapeutic benefits of hP-MSCs by promoting PGE_2_ secretion and M2 macrophage polarization in a colitis model.

The polarization of macrophages is a complex process with multifactor interactions that is regulated by a variety of intracellular signaling molecules and pathways 11. The cytokines secreted by hP-MSCs, such as IGF-1, PGF and PGE_2_, may be involved in the polarization of M2 macrophages 39. PGE_2_, a lipid signaling molecule that supports the expansion of several types of tissue stem cells, is a novel therapeutic target for promoting tissue regeneration *in vivo* 39-41. Macrophages are considered the primary effector cells in regulating tissue repair, and the reprogramming of the macrophage phenotype is mediated through the microenvironment of injured sites 11, 42. These findings suggest that PGE_2_ can not only relieve inflammation but also has significant therapeutic potential for tissue regeneration through macrophages 11. When transplanted into the inflammatory colon, the CS-IGF-1C hydrogel protected the delivered hP-MSCs and further promoted functional and structural recovery of the colon. These benefits can be attributed to the favorable niche produced by the cell-hydrogel interplay, which led to enhanced cell survival, increased secretion of PGE_2_, accelerated M2 macrophage polarization, increased IL-10 expression, and inhibited inflammatory responses.

Recent studies revealed that tissue repair and regeneration are orchestrated by immune responses to tissue damage in the local microenvironment, including networks of cellular components and signaling pathways 11. IL-10 is an essential immunoregulator in the intestinal tract, and IL-10-deficient mice will develop chronic enterocolitis 43, 44. In addition, IL-10-producing macrophages have been shown to suppress acute experimental colitis in mice 45-47. Previous studies have demonstrated that tumor necrosis factor-induced protein 6 (TSG-6) secreted by MSCs could suppress the mucosal inflammatory response in mice with colitis by promoting the production of IL-10 from macrophages 13, 48. In this study, we found that PGE_2_ produced by hP-MSCs mediated the polarization of M2 macrophages, which produced IL-10 to possibly mediate the effect of colon inflammation attenuation.

## Conclusion

Local application of CS-IGF-1C hydrogel with hP-MSCs significantly ameliorates mouse colitis via PGE_2_, which polarizes M2 macrophages and upregulates IL-10 secretion. This approach provides a novel avenue for the effective application of hP-MSC transplantation in IBD treatment. MSCs have been considered an attractive therapeutic strategy for the efficient and safe management of human IBD. Although the underlying mechanisms still require further clarification, the impact of macrophage polarization toward the anti-inflammatory M2 phenotype seems to be especially relevant.

## Abbreviations

BLI: bioluminescence imaging
Chitosan: CS
CS: chitosan
CS-IGF-1C: chitosan-based injectable hydrogel with immobilized the C domain peptide of IGF-1
DAI: disease activity index
Fluc: firefly luciferase
GFP: green fluorescence protein
HGF: hepatocyte grow factor
hP-MSCs: human placenta-derived mesenchymal stem cells
IBD: inflammatory bowel disease
IDO: indoleamine 2,3-dioxygenase
IGF-1C: C domain peptide of insulin-like growth factor-1
IL: interleukin
iNOS: inducible nitric oxide synthase
MSCs: mesenchymal stem cells
MSCs: Mesenchymal stem cells
NO: nitric oxide
PGE_2_: prostaglandin E_2_
ROS: reactive oxygen species
TGF-β1: transforming growth factor-β1
TNBS: trinitrobenzene sulfonic acid
VEGF: vascular endothelial growth factor
