# Supplementary material for: IGF-1C hydrogel improves the therapeutic effects of MSCs on colitis in mice through PGE2-mediated M2 macrophage polarization
Source: Theranostics. 2020 Jun 19;10(17):7697–709. doi: 10.7150/thno.45434 (PMC7359093; doi:10.7150/thno.45434)
Supplement: Supplementary file 1 — Supplementary figures and tables. [file thnov10p7697s1.pdf]

SUPPLEMENTAL DATA

Table S1: Primers used for real-time PCR

Transcription	Prime sequences
hPDGF	Forward: GACTCAGGCGGAATCCAACC
	Reverse: GCCTCCTTAGATCACAGCTCC
hVEGF	Forward: AGGGCAGAATCATCACGAAGT
	Reverse: AGGGTCTCGATTGGATGGCA
hEGF	Forward: TGTCCACGCAATGTGTCTGAA
	Reverse: CATTATCGGGTGAGGAACAACC
hPGF	Forward: GAACGGCTCGTCAGAGGTG
	Reverse: ACAGTGCAGATTCTCATCGCC
hHGF	Forward: GCTATCGGGGTAAAGACCTACA
	Reverse: CGTAGCGTACCTCTGGATTGC
hIGF-1	Forward: GCTCTTCAGTTCGTGTGTGGA
	Reverse: GCCTCCTTAGATCACAGCTCC
hCOX-1	Forward: CTCTGTGCCTAAAGATTGCCC
	Reverse: GTCTCCATAAATGTGGCCGAG
hCOX-2	Forward: CTGGCGCTCAGCCATACAG
	Reverse: CGCACTTATACTGGTCAAATCCC
mIL-10	Forward: CTTACTGACTGGCATGAGGATCA
	Reverse: GCAGCTCTAGGAGCATGTGG
mIL-1β	Forward: GAAATGCCACCTTTTGACAGTG
	Reverse: TGGATGCTCTCATCAGGACAG
mIL-6	Forward: CTGCAAGAGACTTCCATCCAG
	Reverse: AGTGGTATAGACAGGTCTGTTGG
mIFN-γ	Forward: ACAGCAAGGCGAAAAAGGATG
	Reverse: TGGTGGACCACTCGGATGA
mTNF-α	Forward: CTGAACTTCGGGGTGATCGG
	Reverse: GGCTTGTCACTCGAATTTTGAGA
mCD206	Forward: GATACCTGCGACAGTAAACGA
	Reverse: CTGGCTATAAGGGAATTGTGAAG
mCOX-1	Forward: ATGAGTCGAAGGAGTCTCTCG
	Reverse: GCACGGATAGTAACAACAGGGA
mCOX-2	Forward: TTCCAATCCATGTCAAAACCGT
	Reverse: AGTCCGGGTACAGTCACACTT
mOccludin	Forward: TGAAAGTCCACCTCCTTACAGA
	Reverse: CCGGATAAAAAGAGTACGCTGG
mClaudin	Forward: TGCCCCAGTGGAAGATTTACT
	Reverse: CTTTGCGAAACGCAGGACAT
mZO-1	Forward: GCTTTAGCGAACAGAAGGAGC
	Reverse: TTCATTTTTCCGAGACTTCACCA
mGAPDH	Forward: TTGTCTCCTGCGACTTCAAC
	Reverse: GTCATACCAGGAAATGAGCTTG
hGAPDH	Forward: GGAGCGAGATCCCTCCAAAAT
	Reverse: GGCTGTTGTCATACTTCTCATGG

Supplemental Figures & Legends:

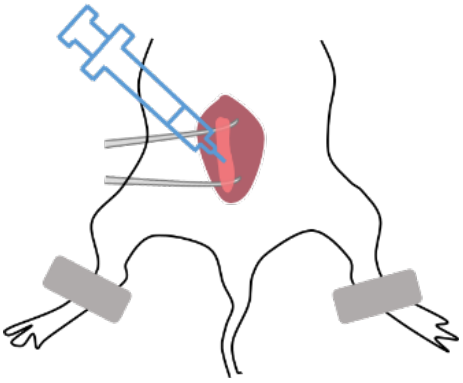

Figure S1. The schematic illustration of laparotomy.

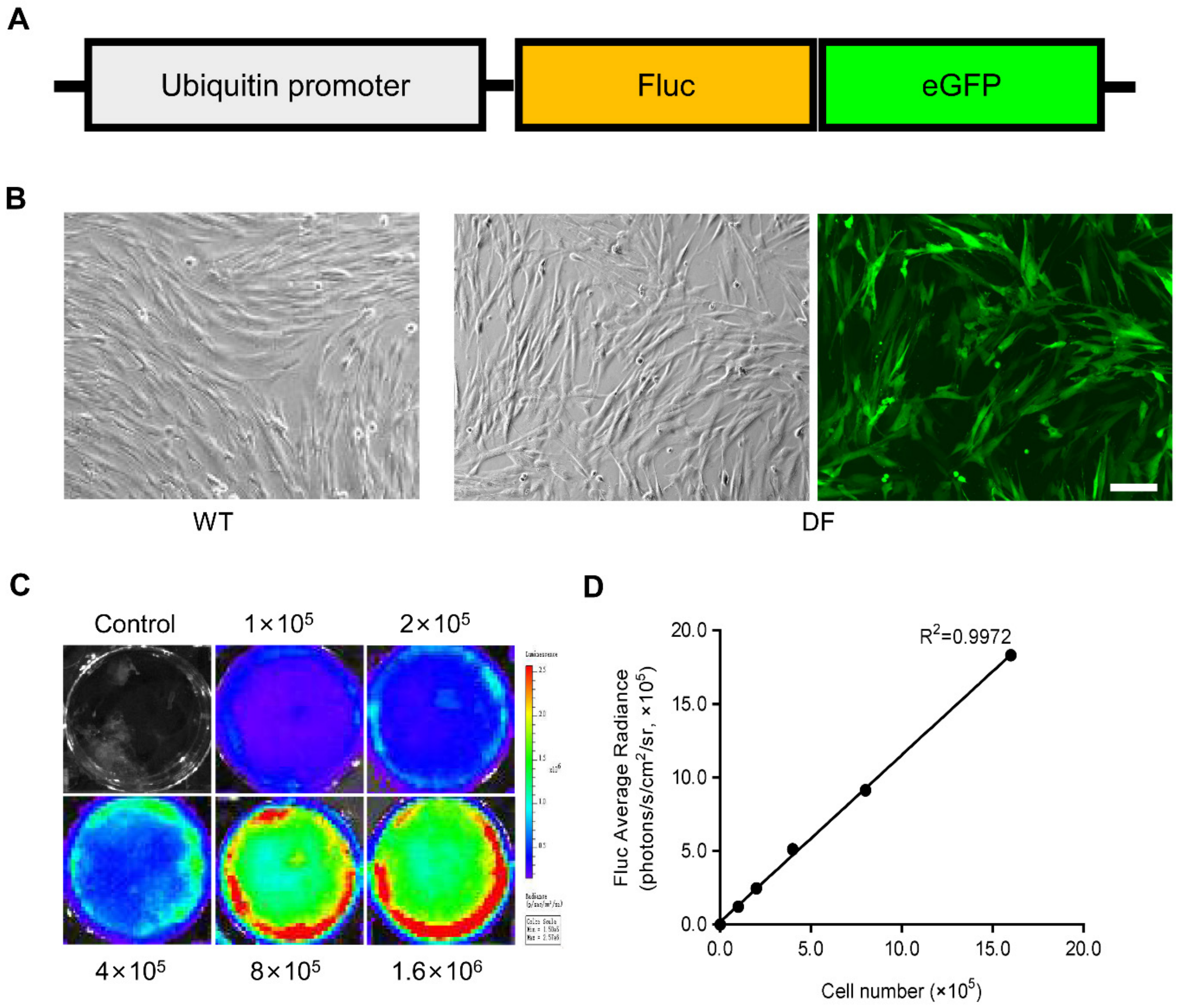

Figure S2. Transduction of hP-MSCs with double fusion (DF) reporter genes. (A) Schematic of the DF reporter gene containing Fluc and eGFP driven by an ubiquitin

promoter. **(B)** Images of untransfected hP-MSCs (WT) and transduced hP-MSCs (DF). Transduced hP-MSCs are strongly positive for eGFP on fluorescence microscopy. **(C)** Bioluminescence imaging of transduced hP-MSCs. **(D)** Analysis of stably transduced hP-MSCs shows a robust correlation between cell numbers and Fluc reporter gene activity. Scale bars, 100 μm.

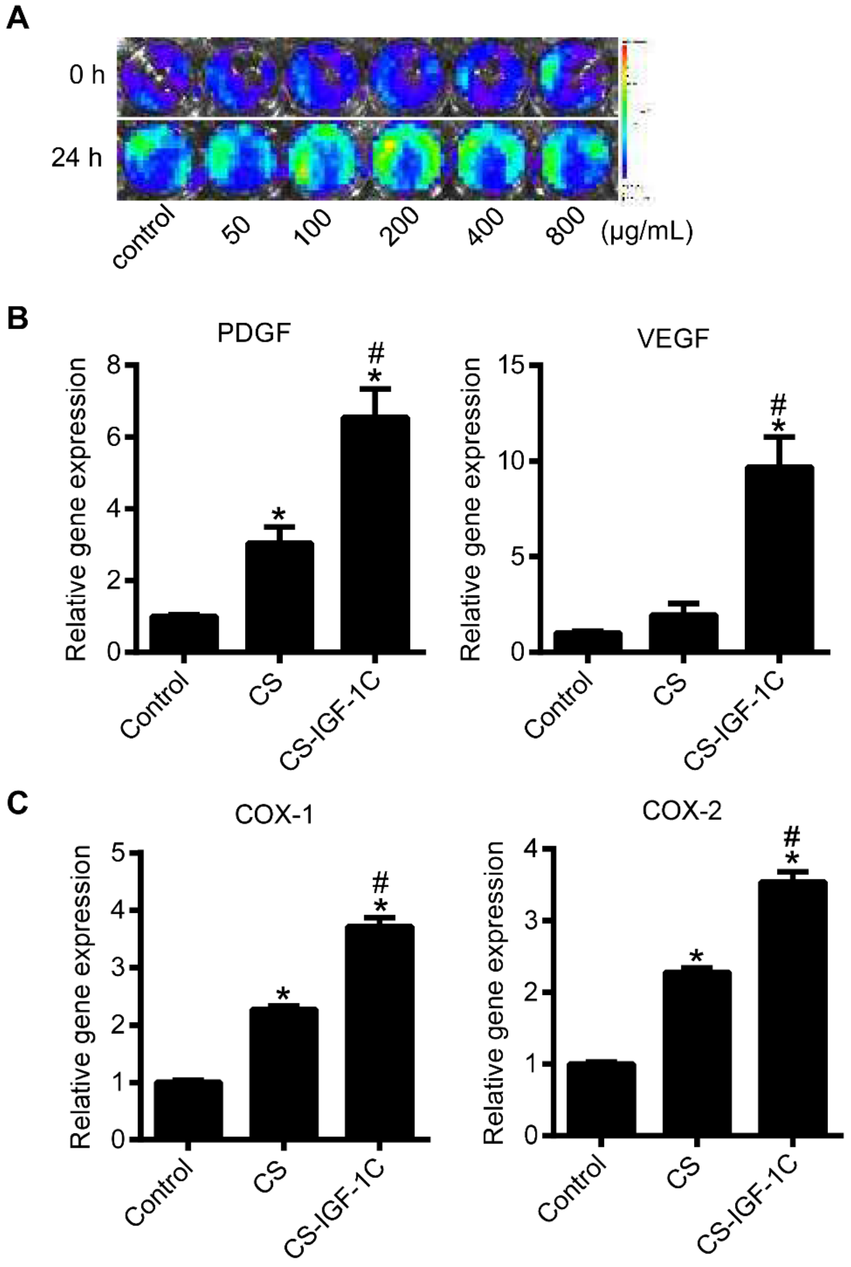

Figure S3. Biocompatibility of CS-IGF-1C hydrogel. (A) Cell proliferation assay of hP-MSCs under different concentrations of CS-IGF-1C hydrogel. **(B)** RT-PCR detected the

relative gene levels of PDGF and VEGF. **(C)** RT-PCR analysis of PGE_2-related gene expression (COX-1 and COX-2). Data are expressed as the mean ± SD. $^{*}P<0.05$ versus hP-MSCs; $^{\#}P<0.05$ versus CS.

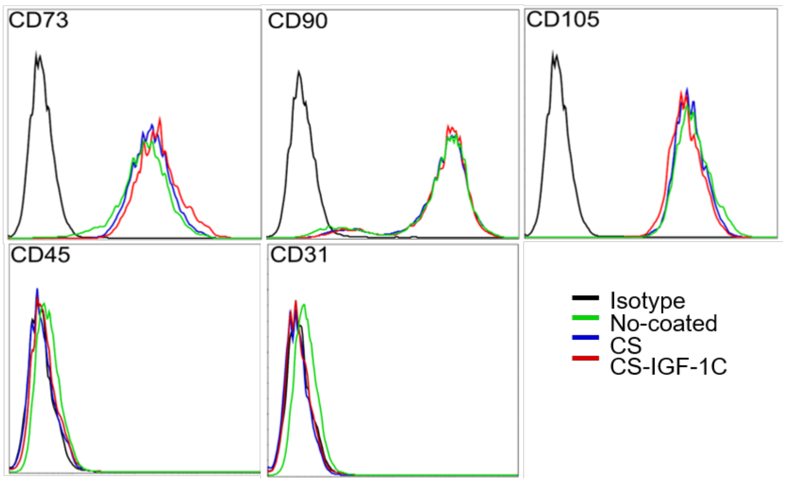

Figure S4. Influence of hydrogels on phenotype of hP-MSCs. The phenotypic profiling of hP-MSCs was assessed by flow cytometry. hP-MSCs were cultured on noncoated, chitosan hydrogel coated, and CS-IGF-1C hydrogel coated plates for 3 days.

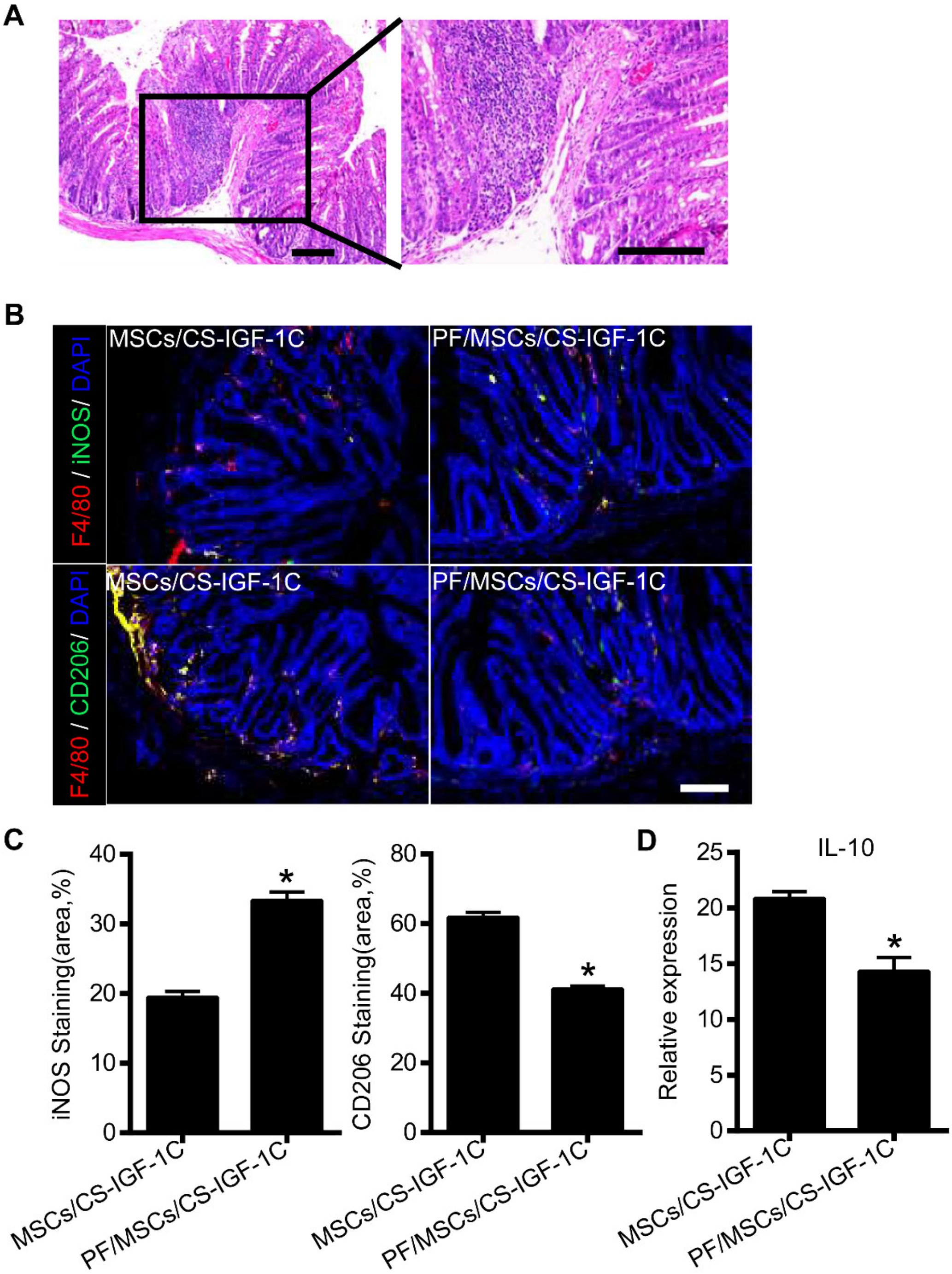

Figure S5. The effect of PGE$_2$-deficient hP-MSCs/CS-IGF-1C cotransplantation on the colitis model. (A) Representative histological sections of mouse colons stained with H&E. Magnification 10 x and 20 x. Scale bar, 100 μm, 50 μm. **(B)** Representative images show iNOS/CD206 (green) and F4/80 (red) immunostaining on day 3 after treatment. **(C)** Quantitative analysis of iNOS-/CD206- positive cells. (**D**) Real-time PCR analysis of IL-10 expression in the colon on day 3 after treatment. Data are expressed as the mean ± SD, n=10.

*$P<0.05$ hP-MSCs/CS-IGF-1C. Scale bar, 100 μm. hP-MSCs/CS-IGF-1C, hP-MSCs cotransplanted with CS-IGF-1C hydrogel. PF/hP-MSCs/CS-IGF-1C, hP-MSCs cotransplanted with CS-IGF-1C hydrogel and EP2 inhibitor. All experiments were performed in triplicate.

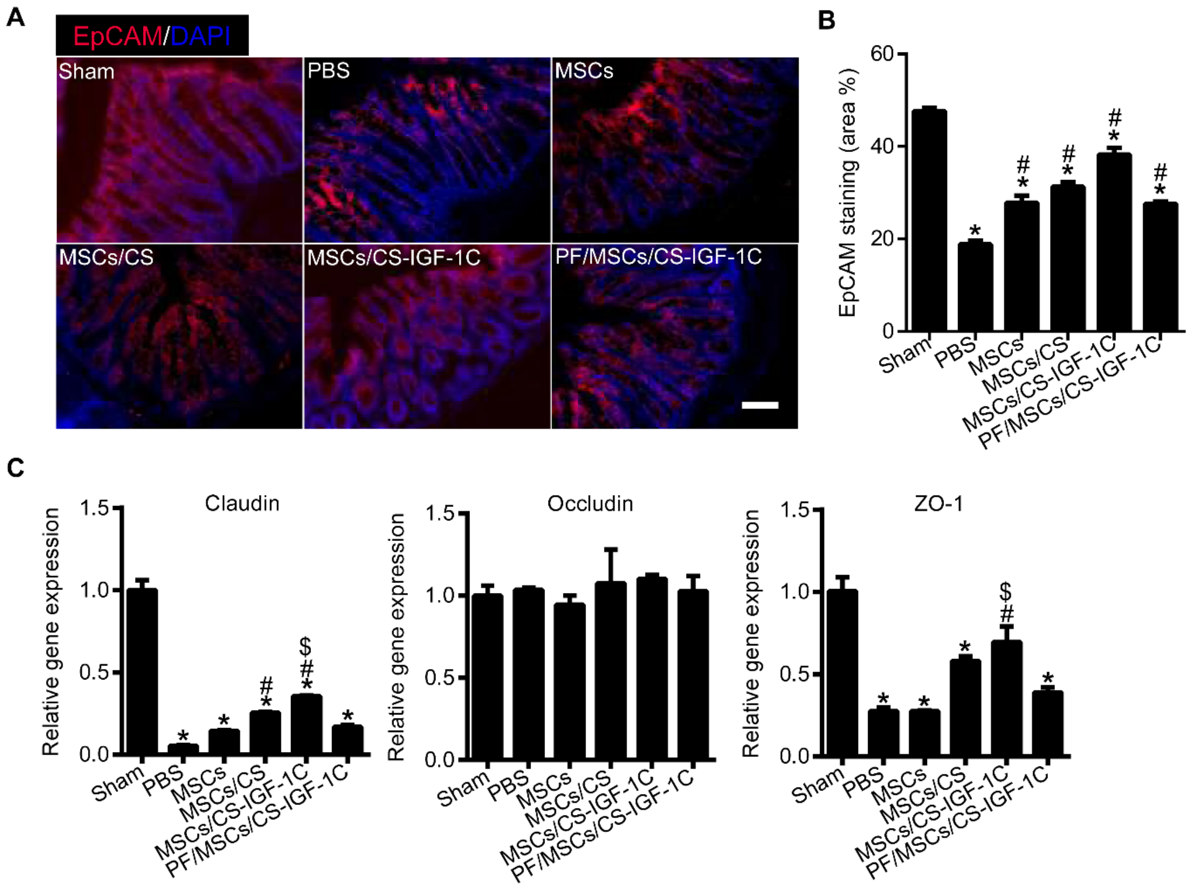

Figure S6. The CS-IGF-1C hydrogel promoted the integrity of intestinal epithelial cells. (A) Representative images show EpCAM immunostaining on day 3 after treatment. **(B)** Quantitative analysis of the EpCAM staining area. **(C)** Real-time PCR analysis of tight junction protein-related gene expression (Claudin, Occludin, and ZO-1) in the colon on day 3 after treatment. Data are expressed as the mean ± SD, n=10. *$P<0.05$ versus sham; #$P<0.05$ versus PBS; $\$P<0.05$ versus hP-MSCs. Magnification 20 x, Scale bar, 100 μm. hP-MSCs/CS, hP-MSCs cotransplanted with CS hydrogel; hP-MSCs/CS-IGF-1C, hP-MSCs cotransplanted with CS-IGF-1C hydrogel. PF/hP-MSCs/CS-IGF-1C, hP-MSCs cotransplanted with CS-IGF-1C hydrogel and EP2 inhibitor. All experiments were

performed in triplicate.

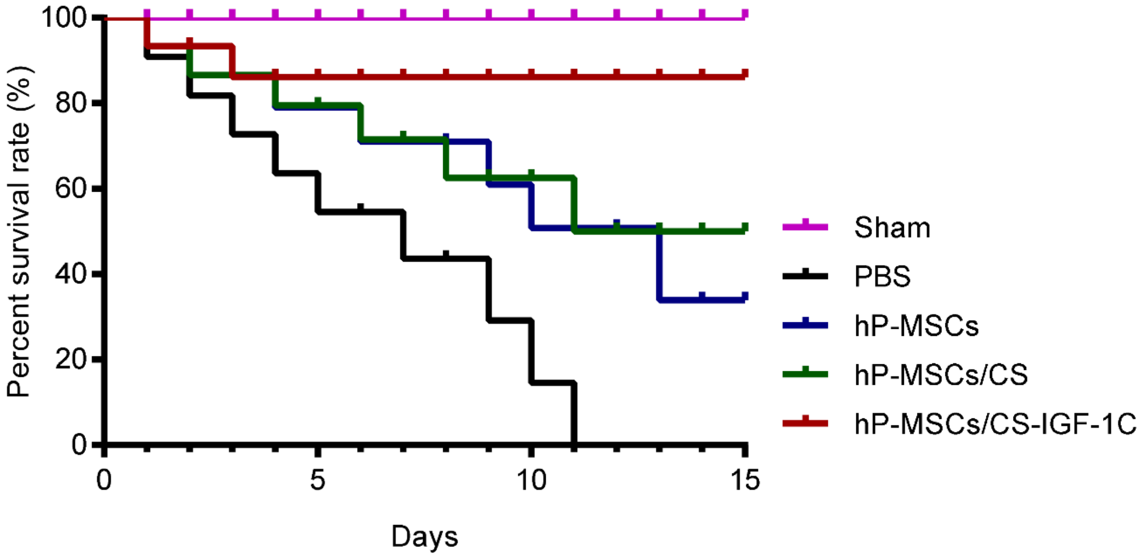

Figure S7. The survival rate of mice that received different treatments. The survival rate of TNBS-induced colitis mice that received different treatments (only hP-MSCs, hP-MSCs with CS hydrogel and hP-MSCs with CS-IGF-1C hydrogel). The number of surviving mice was counted on day 15. n=10.
